# Exploration of Energy Storage Materials for Water Desalination via Next-Generation Capacitive Deionization

**DOI:** 10.3389/fchem.2020.00415

**Published:** 2020-05-15

**Authors:** Wenhui Shi, Xinlong Gao, Jing Mao, Xin Qian, Wenxian Liu, Fangfang Wu, Haibo Li, Zhiyuan Zeng, Jiangnan Shen, Xiehong Cao

**Affiliations:** ^1^Center for Membrane Separation and Water Science & Technology, Ocean College, Zhejiang University of Technology, Hangzhou, China; ^2^College of Materials Science and Engineering, Zhejiang University of Technology, Hangzhou, China; ^3^Ningxia Key Lab Photovolta Material, Ningxia University, Yinchuan, China; ^4^Department of Materials Science and Engineering, City University of Hong Kong, Hong Kong, China

**Keywords:** energy storage, sodium-ion battery, capacitive deionization, water desalination, faradaic electrode

## Abstract

Clean energy and environmental protection are critical to the sustainable development of human society. The numerous emerged electrode materials for energy storage devices offer opportunities for the development of capacitive deionization (CDI), which is considered as a promising water treatment technology with advantages of low cost, high energy efficiency, and wide application. Conventional CDI based on porous carbon electrode has low salt removal capacity which limits its application in high salinity brine. Recently, the faradaic electrode materials inspired by the researches of sodium-batteries appear to be attractive candidates for next-generation CDI which capture ions by the intercalation or redox reactions in the bulk of electrode. In this mini review, we summarize the recent advances in the development of various faradaic materials as CDI electrodes with the discussion of possible strategies to address the problems present.

## Introduction

Clean energy and environmental protection are critical to the sustainable development of human society (Panwar et al., 2011; Shahzad et al., 2017; Liu et al., 2019a). Over the past decades, great efforts have been devoted to the development of various electrochemical energy storage devices, such as lithium-ion batteries sodium-ion batteries (SIBs) (Zhang et al., 2018; Zubi et al., 2018), SIBs (Chao et al., 2018; Delmas, 2018; Fang et al., 2018), metal-air batteries (Zhang X. et al., 2016), and supercapacitors (Raza et al., 2018; Xu et al., 2018; Han Y. et al., 2019). On the other hand, water desalination has played an essential role in resolving the water crisis. Compared with other existing desalination technologies, capacitive deionization (CDI) has the key advantages of low cost, high energy efficiency, easy regeneration, and wide application (Suss et al., 2015). Beyond the desalination of brackish water, CDI has been also applied in wastewater remediation (Wang Z. J. et al., 2017), water softening (Wang and Lin, 2019), the removal of heavy metals (Huang et al., 2016) and organic pollutants (Bharath et al., 2017), as well as ion separation (Shi et al., 2019a).

The concept of CDI was reported as early as 1960s, but the term of CDI was proposed by Farmer et al. until 1996 (Farmer et al., 1996). In recent decades, the research interests in CDI have exponentially grown with the advances of novel electrode materials, cell configurations, and better understanding of ion electrosorption behaviors (Oladunni et al., 2018; Tang et al., 2019). CDI has many similarities with electrochemical energy storage systems, especially supercapacitors, sodium ion batteries (Kim et al., 2014; Fang et al., 2019), etc. These two fields can find common ground in related researches, such as the design of electrode materials, interfacial electrochemistry, and the ion diffusion process in micro-/nano-structured materials (Shi et al., 2019c).

**Figure 1:**
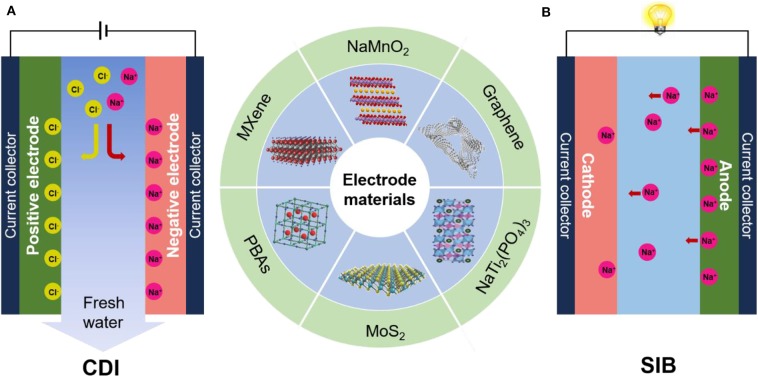
**(A)** Schematic diagram of capacitive deionization (CDI) structure. **(B)** Schematic diagram of Sodium-ion battery (SIB) structure.

As shown in Figure 1A, CDI cells are typically composed of two parallel porous electrodes with a channel in between for water flow. The most widely used electrode for traditional CDI is carbon materials. When a voltage difference of generally ~1 V is applied across the CDI cell, ions are capacitively stored in the porous electrode by forming electrical double layers (EDLs), thereby removing the salt ions from the feed water. When the external power supply is removed or the polarity is reversed, the electrode can be regenerated with the adsorbed ions released to the brine. It is worth mentioning that recently some newly developed configurations of CDI cells have adopted faradaic electrode materials used in batteries, such as hybrid CDI (Lee et al., 2014). Differing from carbon electrodes, faradaic materials store ions by intercalation or redox reactions (inset in Figure 1A) (Pasta et al., 2012). The desalination/regeneration process of CDI is very similar to the charge/discharge process of energy storage devices (Figure 1B). In this mini review, we briefly introduce the key features of CDI and aqueous electrochemical energy storage devices in common, in terms of principle and design strategy of electrode materials. In particular, possible strategies and emerging trends to address the challenges present in CDI were discussed. This review may provide a new insight to the development of novel faradaic electrode materials for the next-generation CDI system.

## Carbon Materials

CDI shares a lot of electrode materials with electrochemical energy storage devices. The CDI and energy storage performances of the representative electrode materials are summarized in Table 1. Among these materials, carbonaceous materials have been widely used in electrochemical sodium storage devices, such as SIBs and sodium ion capacitors (Balogun et al., 2016). They are also the most commonly explored electrodes for conventional CDI, which is based on the EDLs effect of carbon materials to capture ions from salty water (Noonan et al., 2018). Carbon electrode-based CDI has a desalination capacity of about 0.7–14.3 mg g^−1^ (Porada et al., 2013). Recently developed nano-carbon electrodes, such as graphene (Xu et al., 2015a; Zhang et al., 2019), carbon with functional groups (Gao et al., 2016), and hierarchical carbon materials (Gao et al., 2015), can increase the desalination capacity up to 57.13 mg g^−1^ (Li et al., 2017).

**Table 1:** Performance comparison of the same material in CDI and SIB applications, respectively.

**Electrode materials**	**CDI performance**	**References**	**Energy storage performance**	**References**
Graphene	4.29 mg g^−1^ at 2 V in 27 mg L^−1^ NaCl	Li et al., 2010	50 mAh g^−1^ at 6 A g^−1^ in 1 M Na_2_SO_4_ 90% after 1,200 cycles at 6 A g^−1^	Li et al., 2012
Meso-porous rGO nanosheet-assembled 3D graphene	17.1 mg g^−1^ at 1.6 V in 500 mg L^−1^ NaCl	Shi et al., 2016	55.5 mAh g^−1^ at 0.5 A g^−1^ in 1 M Na_2_SO_4_	Choi et al., 2012
MOF-derived porous carbon	13.86 mg g^−1^ at 1.2 V in 500 mg L^−1^ NaCl	Liu et al., 2015	109.7 mAh g^−1^ at 0.2 A g^−1^ in 500 mg L^−1^ NaCl	Wang Z. et al., 2016
Nitrogen-doped graphene sponge	21.0 mg g^−1^ at 1.2 V in 500 mg L^−1^ NaCl ~100% after 30 cycles at 1.2 V	Xu et al., 2015b	154 mAh g^−1^ at 15 A g^−1^ in 1 M NaClO_4_ 99% after 7,000 cycles at 5 A g^−1^	Wang S. Q. et al., 2016
Surface modified carbon	16.7 mg g^−1^ at 1 V in 30 mM NaCl	Gao et al., 2016	30.27 mAh g^−1^ at 1.25 A g^−1^ in 2 M Na_2_SO_4_	Lang et al., 2012
Na_4_Mn_9_O_18_	31.2 mg g^−1^ at 1.2 V in 50 mM NaCl	Lee et al., 2014	39 mAh g^−1^ at 0.2 A g^−1^ in 0.5 M Na_2_SO_4_	Kim et al., 2013
NaTi_2_(PO_4_)_3_/rGO	120 mg g^−1^ at 100 mA g^−1^ in 1,000 mg L^−1^ NaCl 85.7% after 100 cycles at 100 mA g^−1^	Dong et al., 2017	58 mAh g^−1^ at 0.5 A g^−1^ in 1 M Na_2_SO_4_ 50% after 50 cycles at 0.5 A g^−1^	Zhang Q. et al., 2016
Na_3_V_2_(PO_4_)_3_@C	137.20 mg g^−1^ at 1.0 V in 100 mM NaCl	Cao J. L. et al., 2019	58.1 mAh g^−1^ at 1 A g^−1^ in 1 M Na_2_SO_4_ 32% after 30 cycles at 1 A g^−1^	Song et al., 2014
Wire Na_3_V_2_(PO_4_)_3_@C	98 mg g^−1^ at 100 mA g^−1^ in 1,000 mg L^−1^ NaCl 79% after 50 cycles at 100 mA g^−1^	Zhao et al., 2018	58.7 mAh g^−1^ at 1 A g^−1^ in 1 M Na_2_SO_4_ 83% after 600 cycles at A g^−1^	Dong et al., 2017
Na_3_V_2_(PO_4_)_3_/graphene hybrid aerogel	107.5 mg g^−1^ at 100 mA g^−1^ in 1,000 mg L^−1^ NaCl 86.7% after 50 cycles at 100 mA g^−1^	Zhao W. Y. et al., 2019	92 mAh g^−1^ at 50 mA g^−1^ in 0.5 M CH_3_COONa 77% after 200 cycles at 50 mA g^−1^	Li et al., 2016
PBAs	100 mg g^−1^ at 2.8 A m^−2^ in 50 mM NaCl 86% after 50 cycles at 5.7 A m^−2^	Kim et al., 2017	80 mAh g^−1^ at 0.5 A g^−1^ in 0.5 M Na_2_SO_4_ 93% after 8,000 cycles at 10 A g^−1^	Wang J. G. et al., 2017
MXene	45 mg g^−1^ at 1.2 V in 10 g L^−1^ NaCl Stable 60 cycles at 1.2 V	Bao et al., 2018	76.2 mAh g^−1^ at 50 mA g^−1^ in 1 M Na_2_SO_4_ 98% after 500 cycles at 0.2 A g^−1^	Zhu et al., 2017
MoS_2_	16.51 mg cm^−3^ at 1.2 V in 400 mM NaCl	Xing et al., 2017	55 mAh g^−1^ at 5 mV s^−1^ in 1 M Na_2_SO_4_ 82% after 1,000 cycles	Pujari et al., 2017
Na_2_FeP_2_O_7_	30.2 mg g^−1^ at 1.2 V in 100 mM NaCl	Kim et al., 2016	58 mAh g^−1^ at 2 mA cm^−2^ in 2 M Na_2_SO_4_ 89% after 30 cycles at 2 mA cm^−2^	Nakamoto et al., 2016
V_2_O_5_	23.6 mg g^−1^ at 100 mA g^−1^ in 600 mM NaCl Stable 100 cycles at 100 mA g^−1^	Lee et al., 2017b	119.1 mAh g^−1^ at 1 A g^−1^ in 1 M Na_2_SO_4_ 89.6% after 10,000 cycles at 20 mV s^−1^	Ghaly et al., 2019
MnO_2_	25.2 mg g^−1^ at 5 A m^−2^ in 0.1 M NaCl	Hand and Cusick, 2017	176 mAh g^−1^ at 1 mA cm^−2^ in 1 M Na_2_SO_4_ 95% after 2,000 cycles at 100 mV s^−1^	Chodankar et al., 2015

CDI performance of graphene was first reported by Li et al. showing a relatively low desalination capacity of 4.29 mg g^−1^ at 2.0 V in 27 mg L^−1^ NaCl solution (Li et al., 2010). Later works found that building three-dimensional (3D) structure is an effective approach to alleviate the agglomeration of graphene sheets and shorten the diffusion distance (Xu et al., 2015a). For example, Shi et al. developed a hierarchical structure with the interconnected macropores within the graphene networks and nanopores on graphene sheets (Shi et al., 2016, 2019b). A high desalination capacity of 17.1 mg g^−1^ was achieved at a cell potential of 1.6 V.

Metal-organic frameworks (MOFs), a new family of porous crystalline materials, have shown great promise as precursors of carbon-based materials. MOF-derived porous carbon materials have attracted much attentions in both energy storage devices and CDI, due to their large specific surface area and tunable microscopic structures (Xu et al., 2016; Shi et al., 2018). MOF-derived porous carbon was studied as the electrode of CDI for the first time by Pan et al. in 2015. Porous carbon obtained by direct carbonization of ZIF-8 exhibited a desalination capacity of 13.86 mg g^−1^ at a cell potential of 1.2 V in 500 mg L^−1^ NaCl solution. Since then, a large amount of MOF-derived carbon and their composites have been developed with further improved performance (Liu et al., 2015; Gao et al., 2019).

In addition, heteroatom doping can improve the wettability and conductivity of electrode materials, which is often adopted as a surface modification approach. Xu et al. synthesized a nitrogen-doped graphene sponge (NGS) as CDI electrode (Xu et al., 2015b). The NGS electrode with high specific surface area and superior electron mobility exhibited an ultrahigh desalination capacity of 21.0 mg g^−1^ in 500 mg L^−1^ NaCl solution. In order to get an insight into the mechanism of surface modification, Landon and co-workers synthesized functionalized carbon electrode and used it as a model to investigate the relationship between chemical surface charge and desalination performance (Gao et al., 2016). It was found that carbon electrodes with the optimized chemical surface charge can extend the working voltage window, thereby significantly increasing the desalination capacity of the CDI electrode.

Up to now, a large variety of investigations on structural design and surface modification of carbon-based materials have been conducted. There are many researchers engaged in the design, development, preparation, modification, and application of carbon materials. Undoubtedly, performance of CDI can be further enhanced via a further understanding of interfacial and kinetic properties of porous carbon materials in future.

## Faradaic Materials

Inspired by the energy storage systems, various faradaic electrode materials have been actively explored for water desalination in recent years. These materials allow the intercalation or redox reactions, which store ions in the bulk of electrode rather than capacitively stored on the surface. Therefore, they are promising for higher salt removal capability compared with conventional carbon materials (Cai et al., 2018).

Sodium manganese oxide, a layered structured material described as Na_x_MnO_2_ (0 < x ≤ 1), has been intensively investigated in SIBs due to their high capacity and low cost. The pioneering work on the application of faradaic electrodes in water desalination was reported in 2012 (Pasta et al., 2012). Pasta et al. proposed a concept of desalination battery which is comprised by a Na_2−x_Mn_5_O_10_ nanorod as positive electrode and Ag/AgCl as negative electrode. However, this system was mainly served as an energy storage device, salt removal only performed at static with a few hundreds of microliters of electrolyte. More recently, a hybrid CDI system was developed by combining a battery electrode (Na_4_Mn_9_O_18_) with a capacitive electrode (activated carbon) demonstrating improved desalination capacity of 31.2 mg g^−1^ as compared to traditional carbon-based CDI system (Lee et al., 2014).

Recently, sodium superionic conductors (NASICONs), such as NaTi_2_(PO_4_)_3_ (NTP) (Huang et al., 2017, 2019; Guo et al., 2018; Wang et al., 2018; Zhang et al., 2020) and Na_3_V_2_(PO_4_)_3_ (NVP) (Cao J. L. et al., 2019; Cao X. X. et al., 2019), have been considered as promising cathode materials for sodium ion battery due to their high theoretical specific capacity and high Na^+^ conductivity. Yang and co-workers fabricated a hybrid CDI cell with NaTi_2_(PO_4_)_3_/rGO as positive electrode and activated carbon (AC) as negative electrode. During the desalination process, sodium ions are inserted into the NTP electrode, while chloride ions are adsorbed on the EDLs of AC electrode. An ultrahigh desalination capacity of 140 mg g^−1^ in the first cycle was achieved at a current density of 100 mA g^−1^, decreasing to 120 mg g^−1^ after 100 cycle.

Na_3_V_2_(PO_4_)_3_ exhibits a high theoretical capacity (117.6 mAh g^−1^) and good thermal stability. Cao et al. applied Na_3_V_2_(PO_4_)_3_@C as a novel faradaic electrode in a hybrid CDI system showing a desalination capacity of 137.30 mg g^−1^ at a constant voltage of 1.0 V in 100 mM NaCl solution (Cao J. L. et al., 2019). Similar work was reported by Yang and co-workers, who synthesized a conductive polydopamine coated NVP (NVP@C) (Zhao et al., 2018). A faradaic CDI system was fabricated by coupling NVP@C as Na storage electrode and AgCl as Cl storage electrode via conversion reactions. Soon after, they reported a binder-free electrode for CDI, which is prepared from Na_3_V_2_(PO_4_)_3_/graphene hybrid aerogel (Zhao W. Y. et al., 2019).

Due to different sodium capture process of battery electrode as compared to carbon-based materials, battery electrode-based CDI can achieve higher desalination capacity and is also suitable to salty water with high concentration. Another distinct advantage of using battery electrode for CDI is that the co-ion effects of carbon electrode can be eliminated. Therefore, ion-exchange-membrane-free CDI can be achieved which are more compact and possible for miniature-size design. It is worth to mention that composite materials may be considered in future research. Based on the studies of SIBs, reasonable composite materials can improve the efficiency of ion and electron transport (Xu et al., 2017; Cai et al., 2018; Zhou et al., 2018). This strategy may be applied to enhance salt removal performance in CDI. Especially, some composite electrodes may be capable of combining the faradaic and EDLs adsorption principles to increase the amount of salt removal (Yue et al., 2019a). In addition, the concept of integrated and/or self-supported electrodes in SIBs can also be applied to the electrode design of CDI, which makes the preparation process simple and is possible for large-scale electrode production.

Prussian blue (PB) and its analogs (PBAs) with a general formula of Na_x_M_1_[M_2_(CN)_6_]_y_·nH_2_O (M: transition metal ions) are a family of coordination compounds with open framework structure. They have been widely investigated as alternative host materials in sodium ion capacitors, SIBs, and zinc-ion batteries in recent years (Qian et al., 2018). Benefiting from their unique large ionic channels and facile preparation, PB-based materials are promising electrode materials for CDI.

Lee et al. first designed a novel desalination system consisting two PBAs electrodes, i.e., the Na_x_Ni[Fe(CN)_6_] and Na_x_Fe[Fe(CN)_6_] electrodes with an anion exchange membrane in between. Based on the working principle of rocking chair battery, this system achieved efficient desalination during charging and discharging, with a desalination capacity of 59.9 mg g^−1^ (Lee et al., 2017a).

Soon after, Guo et al. assembled a hybrid CDI cell using FeFe(CN)_6_ crystals as positive electrode and AC as negative electrode. The PB crystals prepared by a simple chemical precipitation method undergo reversible Na^+^ insertion/extraction process as the following redox reaction.

$$\begin{array}{l} xNa^{+}+xe^{-}+FeFe{\left(CN\right)}_{6}\;↔\;Na_{x}FeFe{\left(CN\right)}_{6} \end{array}$$

The hybrid CDI cell delivered a high removal capacity of 101.7 mg g^−1^ at a current density of 0.125 mA g^−1^ and good cycling stability (Guo et al., 2017).

Later, Choi et al. conducted a similar study to synthesize a CuFe-PBA framework (K_0.03_Cu[Fe(CN)_6_]_0.65_·0.43H_2_O) which is coupled with porous carbon in a hybrid CDI cell. Besides Na^+^ ions, effective removal of various cations such as K^+^, Mg^2+^, and Ca^2+^ was also demonstrated, showing potential application toward desalination of complex feed waters (Choi et al., 2018).

Along with the exploration of new PB crystals, various strategies of structural and morphological modifications have also been developed to further improve their CDI performance. For example, to enhance the electronic conductivity of the PBs, Vafakhah et al. reported a PB/rGO aerogel by embedding PB nanocubes in rGO 3D networks and applied them in a hybrid CDI system. Compared with pristine PB particles, the PB/rGO composite exhibited a noticeably higher desalination capacity of 130 mg g^−1^ at the current density of 100 mA g^−1^ and higher rate capability. At the same time, the reversible intercalation/de-intercalation mechanism of PB-based electrode was further confirmed by *in-situ* XRD analysis (Vafakhah et al., 2019). In addition, Zhao et al. designed a core–shell structured CuFe-PBA@NiFe-PBA, which combines the high capacity of CuFe-PBA core with highly stable NiFe-PBA shell. This core-shelled PBA material delivered 71.8 ± 2.0 mg g^−1^ at 0.5 mA cm^−2^, which is over 32% higher than those of CuFe-PBA and NiFe-PBA, respectively (Zhao Y. B. et al., 2019).

Although their potential advantages, PB-based materials suffer from unsatisfactory performance which is mainly caused by a large amount of lattice defects and coordinated water. The defects largely reduce the utilization of redox-active sites in the PB framework that allow sodium storage, thus leading to a low salt removal capacity. Therefore, in the future research of PB-based CDI electrodes, efforts can be made to develop high-crystallinity PB electrode materials. In addition, related researches on SIBs have revealed that particle size of PBA crystals has significant influence on ion diffusion rate and the rate performance. This finding is also important for the desalination rate of CDI. Unfortunately, nano-sized PB normally has higher surface defect density, a possible solution is to rationally construct composite electrode with the integrity of crystal structure of PBA maintaining.

The breakthrough of graphene has promoted intensive researches on two-dimensional (2D) nanomaterials in past decades. The emerging new types of 2D nanomaterials and their composites have been widely used in various fields including electrochemical energy storage (Liu et al., 2019b, 2020; Zhao H. W. et al., 2019; Wu et al., 2020). MXene, first reported by Naguib et al., is a family of layer structured metal carbide/nitride which shares a general formula of M_n+1_AX_n_ (M is early transition metal, A is an element of group IIIA and IVA, X is C and/or N). After selective etching of A layers, the exfoliated MXene layer with ultrathin thickness is a promising 2D material (Naguib et al., 2011). Compared with other 2D materials such as graphene, MXene has unique advantages of abundant functional groups and tunable properties owing to various chemical composition and controllable interlayer space (VahidMohammadi et al., 2019). MXenes have been proved promising for efficient sodium storage, especially in SIBs and capacitors, delivering specific capacities of 30–76.2 mAh g^−1^ (Zhu et al., 2017; Guan et al., 2020).

Srimuk et al. first reported the application of MXene in a symmetrical CDI cell prepared by direct casting of Ti_3_C_2_ onto a porous separator without addition of binder. The fabricated CDI cell demonstrated a desalinization capacity of more than 13 mg g^−1^ in 5 mM NaCl solution, which is comparable to most of conventional carbon materials for CDI (Srimuk et al., 2016). The relatively low capacity maybe due to the restacking of MXene sheets.

Later, Bao et al. prepared an aerogel-like porous Ti_3_C_2_T_x_ MXene electrode by vacuum freeze-drying process. Impressively, the porous MXene demonstrated a high volumetric salt removal capacity of 118 mg cm^−3^ which is attributed to effective utilization of the 2D surfaces (Bao et al., 2018). Similarly, Ma et al. prepared a free-standing Ti_3_C_2_T_x_ MXene film as binder-free electrode for CDI (Sriramulu and Yang, 2019). Recently, it was found that the introduction of heteroatom dopants to MXene can effectively improve the electronic conductivity and reduce the risk of restacking as well (Amiri et al., 2020).

As a CDI electrode, MXene has the following competitive advantages. First, MXene has intrinsically high volumetric capacitance, enabling high desalination capacity. Second, MXene is accessible to other types of ions beyond Na, which makes it possible for applications in real feedwater (Lukatskaya et al., 2013; Come et al., 2015). In addition, as a typical 2D layered material, MXene has the common feature of strong attraction between layers. Therefore, it can be used as a binder-free electrode, which has appealing advantage for miniature and portable CDI devices. However, most of etching processes of MXene involve the toxic HF which limits the large-scale preparation of MXene in CDI. In future, the etching method need to be further developed to fulfill the requirements of commercial applications.

Another attractive 2D material is few-layered MoS_2_ sheets or flakes, which have been insensitively studied as anode material for SIBs (Sun et al., 2018). Recently, CDI performance of chemically exfoliated MoS_2_ (ce-MoS_2_) nanosheets were investigated by Cao and co-workers (Xing et al., 2017). The ce-MoS_2_ nanosheets demonstrated a good cycling stability and a desalination capacity of 8.81 mg g^−1^ at 1.2 V in 400 mM NaCl solution. MoS_2_ as CDI electrode cannot give a satisfactory performance, possibly due to the poor conductivity and low accessible active sites. Shortly afterward, various low dimensional nanomaterials such as CNT (Srimuk et al., 2017), graphene (Han J. L. et al., 2019; Peng et al., 2020), or g-C_3_N_4_ (Tian et al., 2020) were employed to further improve the performance of MoS_2_.

MoS_2_ can be obtained from a naturally abundant mineral by a simple exfoliation method, which is a low-cost electrode for CDI. However, it is difficult to control the number of layers of the obtained MoS_2_ sheets, which greatly affects the ion diffusion process and leads to rapid capacity fading. Further work is recommended to investigate the relationship between thickness and desalination performance of MoS_2_.

In addition, other commonly used battery electrode materials have also been applied in CDI, such as Na_2_FeP_2_O_7_ (Kim et al., 2016), multi walled CNT/hV_2_O_5_ composite (Lee et al., 2017b), and MnO_2_ (Hand and Cusick, 2017).

## Summary

In summary, CDI is an emerging water treatment technology which shares many similarities with electrochemical energy storage systems. In the near future, the CDI research community is expected to grow larger with the development of novel electrode materials and cell architectures. Obviously, CDI has many advantages over other desalination technologies, including mild operation conditions with no high pressure and heat source required, simple cell configuration, low-cost and high energy efficiency for low salinity streams. However, it is still facing great challenges of application in real feedwaters. More efforts can be made in future to the investigation of electrode materials capable of capturing multivalent ions. Moreover, the adsorption behavior of soluble organics in water is more complicated, which may cause biofouling and affect the desalination performance and service life of CDI.

Another future direction of CDI technology may be the combination with renewable energy resources, such as solar energy and wind energy, to reduce the energy consumption. For example, a recent work demonstrated a flow-electrode CDI system with low energy consumption (Desai et al., 2018). It is also promising to develop miniaturized and portable desalination devices which may have potential applications in exploration and disaster-affected area.

The adsorption of chloride ions will also affect the desalination performance of the whole device. Although silver has been investigated for Cl^−^ ion storage (Yue et al., 2019b), high cost of silver limits its large-scale application in water treatment. Other types of materials, such as bismuth, may be considered. Moreover, researchers may also pay attention to relevant achievements in the recent developed chloride ion battery (Pasta et al., 2012; Nam and Choi, 2017).

In addition, the salt removal mechanism of many recently developed battery electrode materials is different from the generally accepted EDLs principle. Therefore, combining *in-situ* technology to reveal the correlation between the structure of battery electrode materials and its corresponding desalination performance, as well as the desalination mechanism of novel battery materials is another interesting research direction.

## Author Contributions

WS, XG, JM, XQ, WL, FW, HL, ZZ, JS, and XC contributed to the discussion and writing in this work.

## Conflict of Interest

The authors declare that the research was conducted in the absence of any commercial or financial relationships that could be construed as a potential conflict of interest.
